# Single Molecular Semi-Sliding Ferroelectricity/Multiferroicity

**DOI:** 10.34133/research.0428

**Published:** 2024-08-05

**Authors:** Tingting Zhong, Hong Zhang, Menghao Wu

**Affiliations:** ^1^ Department of Physics, Zhejiang Sci-Tech University, Hangzhou, Zhejiang 310018, China.; ^2^ School of Physics, Huazhong University of Science and Technology, Wuhan, Hubei 430074, China.

## Abstract

In recent years, the unique mechanism of sliding ferroelectricity with ultralow switching barriers has been experimentally verified in a series of 2-dimensional (2D) materials. However, its practical applications are hindered by the low polarizations, the challenges in synthesis of ferroelectric phases limited in specific stacking configurations, and the low density for data storage since the switching process involves large-area simultaneous sliding of a whole layer. Herein, through first-principles calculations, we propose a type of semi-sliding ferroelectricity in the single metal porphyrin molecule intercalated in 2D bilayers. An enhanced vertical polarization can be formed independent on stacking configurations and switched via sliding of the molecule accompanied by the vertical displacements of its metal ion anchored from the upper layer to the lower layer. Such semi-sliding ferroelectricity enables each molecule to store 1 bit data independently, and the density for data storage can be greatly enhanced. When the bilayer exhibits intralayer ferromagnetism and interlayer antiferromagnetic coupling, a considerable difference in Curie temperature between 2 layers and a switchable net magnetization can be formed due to the vertical polarization. At a certain range of temperature, the exchange of paramagnetic–ferromagnetic phases between 2 layers is accompanied by ferroelectric switching, leading to a hitherto unreported type of multiferroic coupling that is long-sought for efficient “magnetic reading + electric writing”.

## Introduction

Ferroelectric materials, with electrically switchable polarizations induced by symmetry breaking in crystal lattice, have been widely used in sensors, actuators, memories, etc. [1]. In particular, the unique sliding ferroelectricity exists in many 2-dimensional (2D) van der Waals bilayers [2] where the inequivalency between upper and lower layers leads to a net interlayer charge transfer. The induced vertical polarization can be electrically switchable simply via interlayer sliding, and the sliding barrier is much lower compared with traditional ferroelectrics, which may greatly reduce the required energy for switching [3]. Within several years, such ferroelectricity have been experimentally verified in bilayer/multilayer boron nitride (BN) [4–6], InSe [7], transition-metal dichalcogenides like WTe_2_ [8–10], MoS_2_ [11–16], and ReS_2_ [17], and even amphidynamic crystal [18]. However, as summarized in the previous review [19], the density for data storage is limited since the switching process involves large-area simultaneous sliding of a whole layer with high in-plane rigidity. Meanwhile, the polarizations are generally weak (<2 pC/m) due to weak interlayer charge transfer. Moreover, such ferroelectricity emerges only in certain stacking configurations, i.e., parallel stacking for BN or MoS_2_ bilayers, and the controlling of their formations in large-scale synthesis is much more challenging compared with antiparallel stacking, also noting that a Moire superlattice with almost compensated polarization upon a nonzero twist angle is generally formed by the tear-and-stack method [4].

To resolve the above issues, in this paper, through first-principles calculations, we predict the existence of semi-sliding ferroelectricity in van der Waals bilayers intercalated by metal porphyrin molecules widely studied previously [20,21]. We note that similar metal porphyrin–MoS_2_ systems have been experimentally explored, and aside from intercalation of bilayer [22,23], the stacking of one layer on another porphyrin-decorated monolayer can also be a feasible approach. Both sliding and vertical displacements of metal ions are involved during switching, giving rise to a moderate barrier, high polarization, and ultrahigh data storage density up to 10^5^ Tbit/inch^2^ as each molecule may store 1 bit data independently. Meanwhile, such ferroelectricity may exist in bilayers of various stacking configurations (parallel, antiparallel, or even with a twist angle). In some magnetic bilayer intercalated by metal porphyrin, such ferroelectric switching can give rise to switching of a net magnetization, rendering the long-sought efficient magnetic reading + electric writing, which is elusive in current multiferroics due to the mutual exclusive origins of magnetism and ferroelectricity [24,25]. We even propose a new type of multiferroicity where the exchange of paraelectric–ferroelectric phases between 2 layers can be achieved by ferroelectric switching, and the switchable magnetic moment can be greatly enhanced.

## Results and Discussion

**Fig. 1.**
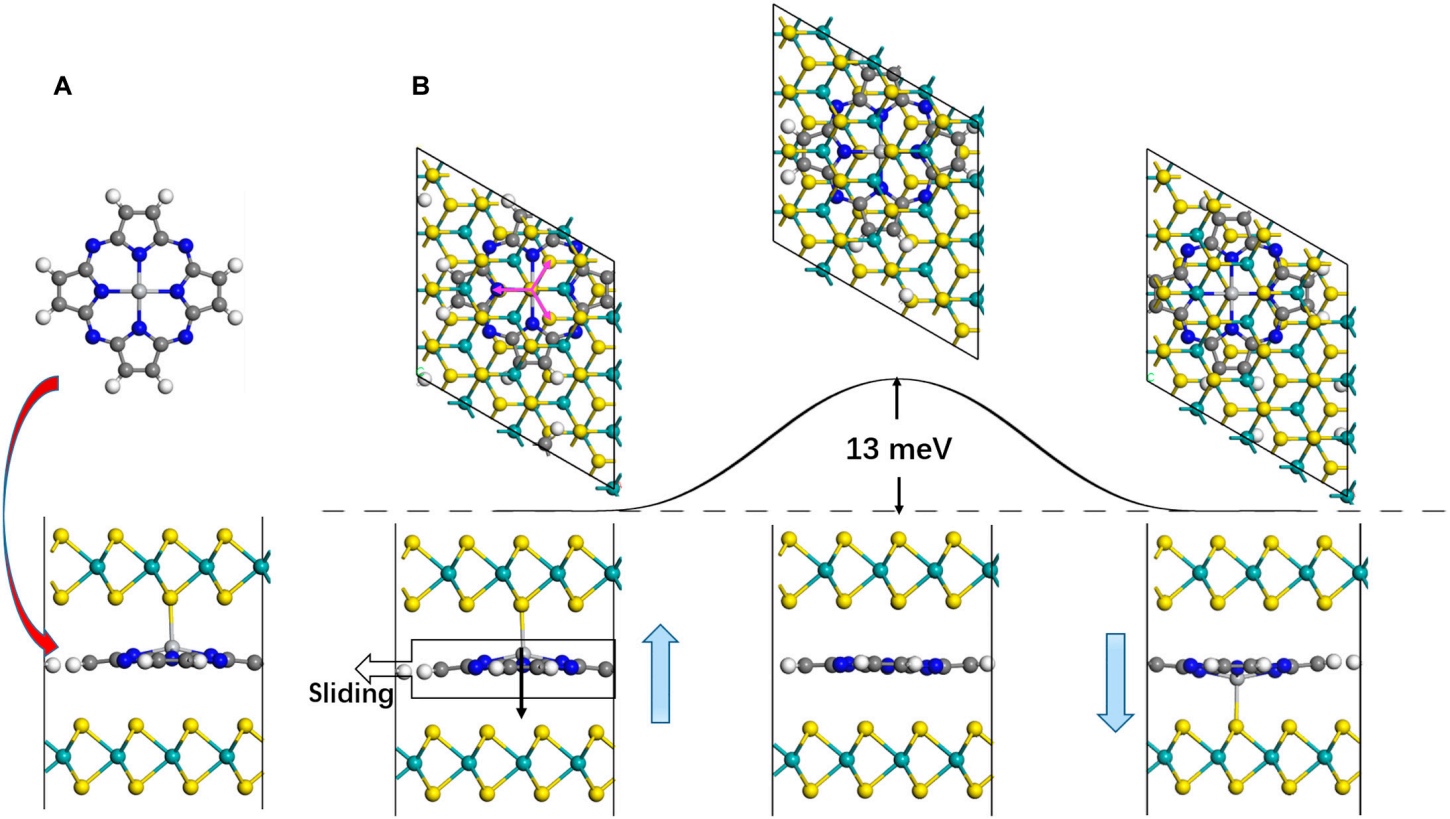
(A) The intercalation of TiP molecules in AB-stacked bilayer MoS_2_, and (B) its ferroelectric switching pathway, where the blue arrows indicate the directions of polarization, and pink arrows denote the in-plane sliding directions.

First we choose titanium porphyrin (TiP) [26,27] as a paradigmatic case, and when a TiP molecule is intercalated between the AB-stacked bilayer MoS_2_, the Ti ion will be inclined to bind with one sulfur anion of one of the MoS_2_ layers, as shown in Fig. 1A, which breaks the symmetry and gives rise to a vertical polarization of 0.27 eÅ per unit cell (around 3.07 pC/m). This value is much enhanced compared with sliding ferroelectricity in pure bilayer BN (~2.08 pC/m) and bilayer MoS_2_ (~0.97 pC/m) [2]. We have checked many configurations, and in our simulations, they are all automatically optimized to the structure in Fig. 1A, which should be the ground state. If the TiP molecules are substituted by MgP or ZnP molecules with metal ions of smaller radius, similar ferroelectricity may still emerge while the polarizations will be greatly reduced (e.g., 0.57 and 0.24 pC/m, respectively, for MgP and ZnP).

To reverse such polarization, the Ti ion need to bind to one nearest S atom of the other layer, which is not directly below it. Herein, the vertical displacement of Ti ion (1.38 Å) as well as the in-plane sliding of the whole molecule (1.81 Å) along either of the marked pink arrows is involved during switching, which is denoted as semi-sliding ferroelectricity. The energy barrier for the ferroelectric switching is estimated to be around 13 meV/atom for each TiP molecule as illustrated in Fig. 1 calculated using the nudged elastic band (NEB) method [28], comparable to the typical barriers of sliding ferroelectricity. Its thermal stability is confirmed by ab initio molecular dynamics (AIMD) simulation (see Fig. S1), where the ferroelectricity revealed by the asymmetric binding of the molecule between 2 layers is robust at 500 K. Thus, each molecule should be able to store 1 bit data independently at ambient conditions, and the upper limit of areal storage density can be enhanced from the previous superparamagnetic limit of ~40 GB/in^2^ to ~10^6^ GB/in^2^.

**Fig. 2.**
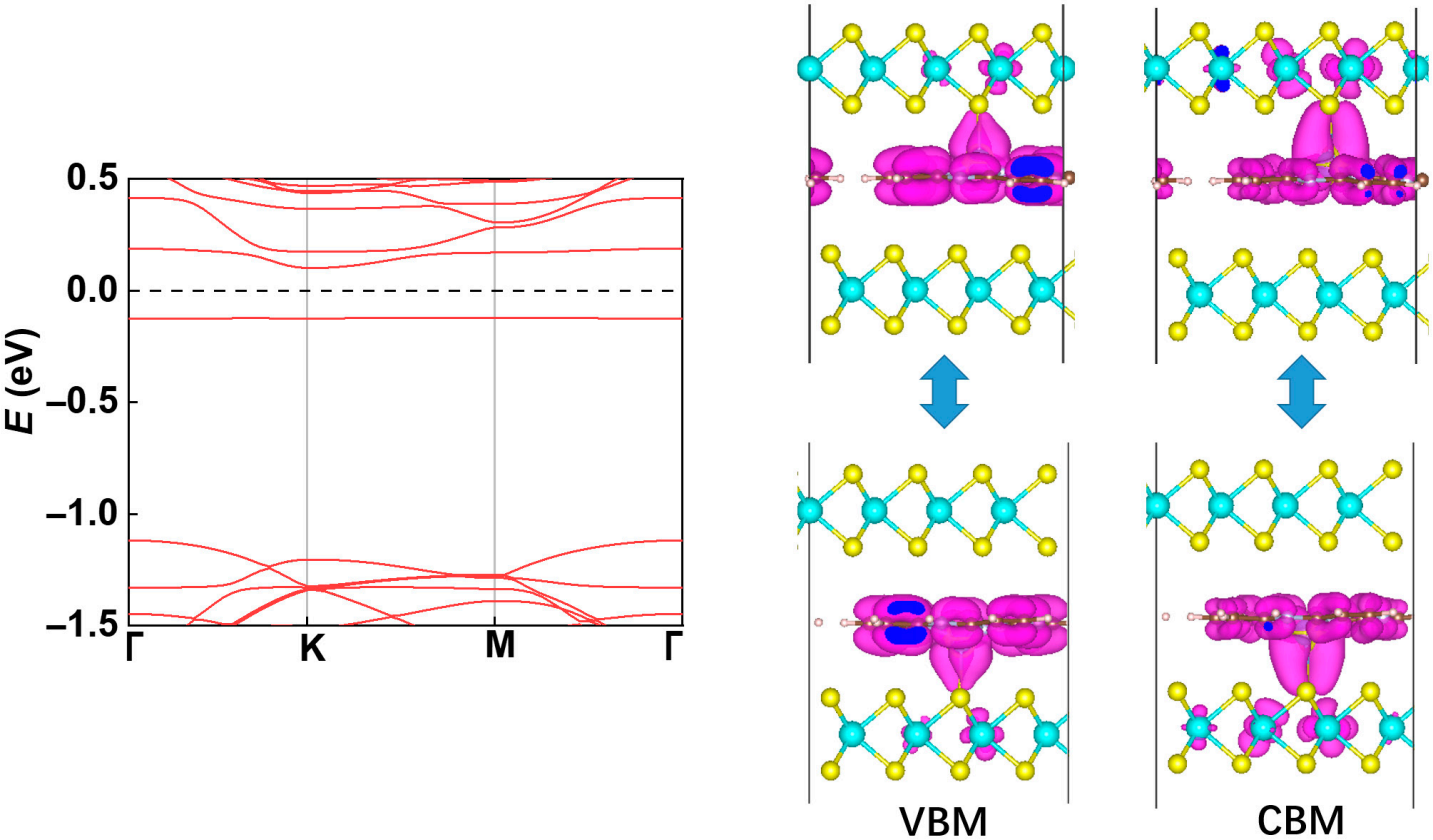
The band structure bilayer MoS_2_ with intercalation of TiP molecules, and the change of VBM and CBM distribution upon ferroelectric switching.

As revealed by the band structure in Fig. 2, the system is a ferroelectric semiconductor [29] with an indirect energy gap of 0.22 eV. In previous studies, the band splitting of AB-stacked bilayer MoS_2_ with sliding ferroelectricity is mostly attributed to the interlayer hybridization, which is much larger compared with the splitting induced by the weak vertical polarization [30]. However, herein the enhanced vertical polarization play a much important role compared with the interlayer hybridization that is weakened by molecule intercalation. As shown in Fig. 2, both the valence band maximum (VBM) and the conduction band minimum (CBM) are mainly distributed in the molecule and its binding MoS_2_ layer (such feature is maintained in our simulation using hybrid functional, as shown in Fig. S2). Upon ferroelectric switching, such distribution will be altered with the binding MoS_2_ layer. If 2 electrodes are attached to the same layer, the ferroelectric switching is likely to be detected by the difference in measured conductance.

**Fig. 3.**
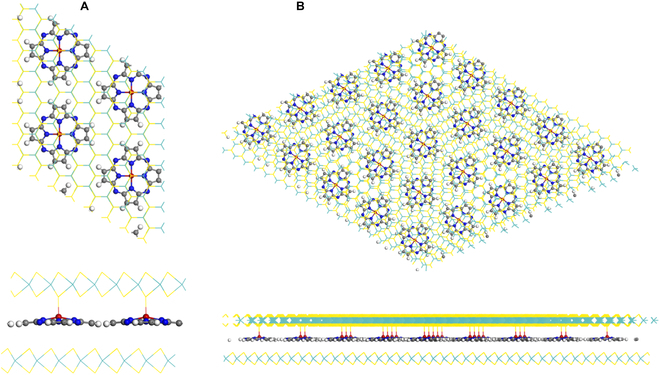
The intercalation of TiP molecules in (A) antiparallel stacking and (B) twisted bilayer MoS_2_, both leading to asymmetric binding and vertical polarizations.

The formation of semi-sliding ferroelectricity seems to be independent on the stacking configuration of bilayers, which is distinct from sliding ferroelectricity. For example, only parallel-stacking BN and MoS_2_ bilayer are ferroelectric, while they are non-polar in antiparallel stacking configuration. Currently, either the controllable synthesis of the ferroelectric configurations or their formation using the “tear-and-stack” method without twist angle remains elusive. However, even when the MoS_2_ bilayer is antiparallel stacked, or with a twist angle, as displayed in Fig. 3, vertical polarizations can still be formed as each Ti ion of TiP will bind with one sulfur anion of one of the MoS_2_ layers, which can be reversed via vertical displacement of the Ti ion combined with the in-plane sliding of the whole molecule.

**Fig. 4.**
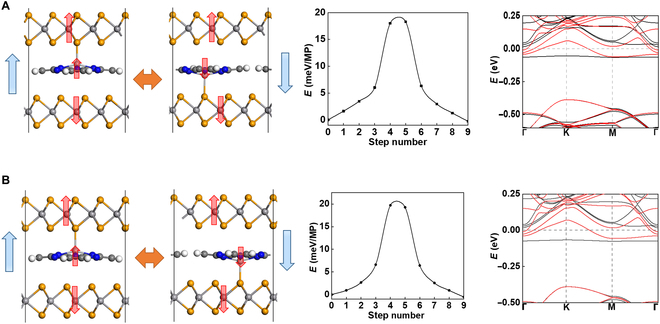
The bi-stable polar states, switching pathway, and band structure of TiP molecules intercalated in (A) parallel and (B) antiparallel stacking bilayer VSe_2_, where the red arrows on vanadium and titanium atoms denote spin directions, and the blue arrows represent the electrical polarization directions.

The coupling between ferroelectricity and magnetism is also expected if molecules like TiP are intercalated between magnetic bilayers, e.g., VSe_2_ bilayer that exhibits intralayer ferromagnetism and interlayer antiferromagnetic coupling according to a previous study [31]. As shown in Fig. 4A, the Ti ion will be inclined to bind with one Se anion of one of the VSe_2_ layers, which gives rise to a reduced vertical polarization around 0.21 pC/m. Similarly, upon sliding of TiP molecules and vertical displacements of the Ti ions, they alternately bind with the surface Se ions of the upper and lower VSe_2_ layers, while the switching barrier is estimated to be over 20 meV/atom for each molecule according to the NEB calculation of pathway. AIMD simulations in Fig. S1 indicate that the ferroelectricity can be robust at 300 K. The system is a spin-polarized metal as revealed by its band structure, and the reduced polarization may be attributed to the enhanced charge screening.

Despite the interlayer antiferromagnetic coupling, the system may still exhibit a net magnetization due to the intercalation of TiP molecules. In the ground state, each Ti ion carries a magnetic moment around 0.2 μ_B_, which is ferromagnetic coupled to the binding VSe_2_ layer, resulting in a net magnetic moment around 0.30 μ_B_. This net magnetic moment can be reversed via polarization switching upon sliding of the TiP molecule, and such coupling between ferroelectricity and magnetism is long-sought for realizing efficient “magnetic reading + electric writing”. Similarly, such semi-sliding multiferroicity still emerges when the bilayer is antiparallel stacking, as well as with a similar switching pathway, barrier, and band structure compared with parallel stacking configuration, as shown in Fig. 4B.

**Fig. 5.**
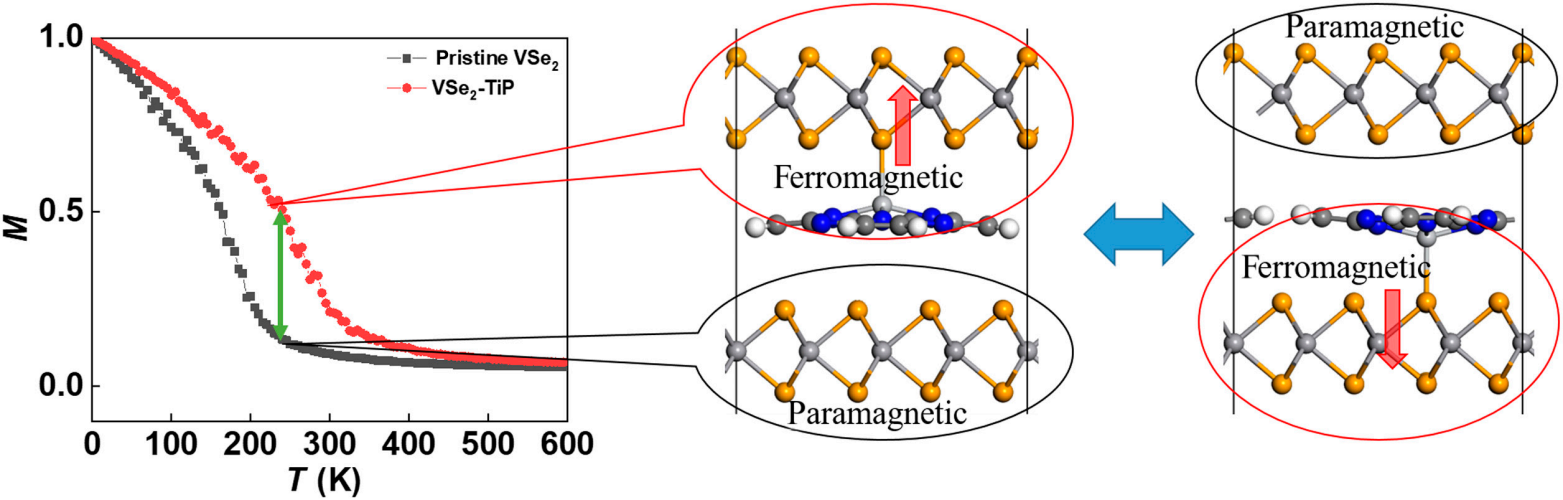
Dependence of the magnetization on temperature for pristine VSe_2_ monolayer and the VSe_2_ layer binding with TiP molecules. For bilayer VSe_2_ intercalated by TiP molecules, at the temperature marked by the green arrow, the exchange of paramagnetic–ferromagnetic phases between 2 layers can be achieved by ferroelectric switching.

It is noteworthy that the switchable net magnetic moment around 0.30 μ_B_ is estimated for the ground state at 0 K. This value can be even greatly enhanced at a certain range of elevated temperature according to our Monte Carlo simulations based on the Heisenberg model:

$$\hat{H}=-\frac{1}{2}\sum_{\langle \mathrm{ij}\rangle }J\vec{S_{i}}\cdot \vec{S_{j}}-\sum_{\langle i\rangle }D{\left(S_{\mathrm{iz}}\right)}^{2}$$ (1)

where *J* is defined as the nearest neighboring exchange coupling parameter, *D* (estimated to be 2.16 meV) is the single-site magnetic anisotropy energy, and |*S*| = 1/2. The calculated *J* values for the VSe_2_ layer binding with TiP molecules and pristine VSe_2_ monolayer are 160.63 meV and 109.38 meV, respectively, where the ferromagnetic coupling seems to be greatly enhanced upon the binding of TiP molecules, giving rise to a much elevated Curie temperature close to room temperature, as displayed in Fig. 5. At a temperature of 240 K marked by the green arrows, the lower layer is close to the paraelectric phase while the upper layer is still ferromagnetic with a magnetization much larger than the TiP molecules. Upon ferroelectric switching, the upper layer becomes paramagnetic while the lower layer becomes ferromagnetic, and the enhanced net magnetization of the system is reversed. The switchable magnetization is maximized at finite temperature (8.2 μ_B_/unit cell around 240 K) instead of 0 K, which goes beyond the previous phenomenological model on magnetic ordering.

## Conclusion

In summary, we propose semi-sliding ferroelectricity in the single metal porphyrin molecule intercalated in 2D bilayers independent on stacking configurations, and the unique switching mode leads to a moderate barrier, high polarization, and ultrahigh data storage density, potentially resolving the major issues of sliding ferroelectricity for practical applications. When the bilayer exhibits intralayer ferromagnetism and interlayer antiferromagnetic coupling, a considerable difference in Curie temperature between 2 layers and a switchable net magnetization can be formed due to the vertical polarization. A new type of multiferroicity may emerge as ferroelectric switching leads to exchange of paramagnetic–ferromagnetic phases between 2 layers, and the switchable magnetic moment can be greatly enhanced. Our prediction does not only exploit new types of ferroelectricity and multiferroic couplings, but also propose a way of resolving current issues for practical applications, which may stimulate further experimental efforts.

## Methods

Our density functional theory calculations were performed using the Vienna Ab initio Simulation Package (VASP 5.4.4) code [32,33].The exchange–correlation effect was described within the generalized gradient approximation in the Perdew–Burke–Ernzerhof [34] functional, and the projector augmented wave [35] method was adopted. For VSe_2_-based systems, we employed effective Coulomb interaction (*U*_eff_) values of 1.2 eV for V-d and 2 eV for Ti-d orbitals to consider on-site Coulomb interaction, following a previous study [31]. The kinetic energy cutoff was set to be 400 eV. In the intercalated bilayer system, to eliminate the interaction between proximate molecules, a vacuum space of 17 Å and a 4 × 4 supercell are adopted so the distance between adjacent molecules exceeds 3.5 Å and their interaction is negligible. The Brillouin zone was sampled using a 3 × 3 × 1 k-point grid in the Monkhorst–Pack scheme [36]. For the geometry optimization, the PBE-D2 functional of Grimme [37] was applied to take into consideration van der Waals interactions. The convergence threshold for self-consistent-field iteration was set to be 10^−5^ eV, and the atomic positions were fully optimized until the forces on all atoms are less than 0.01 eV Å^−1^. Ferroelectric polarizations were computed by the dipole moment correction method, which can give approximately the same value as the experimental measurement in the metallic WTe_2_ bilayer [38]. The ferroelectric switching pathways were calculated by using the climbing image NEB method [28]. In our Monte Carlo simulations, a 2D 20 × 20 supercell with 2 × 10^5^ iterations was adopted, allowing spins at all magnetic sites to flip randomly.

## Data Availability

The conclusions of the study are available in the paper and the Supplementary Materials, and there are no restrictions on data availability.
