# Supplementary material for: Single Molecular Semi-Sliding Ferroelectricity/Multiferroicity
Source: Research (Wash D C). 2024 Aug 5;7:0428. doi: 10.34133/research.0428 (PMC11298321; doi:10.34133/research.0428)
Supplement: Supplementary 1 — Figs. S1 and S2 [file research.0428.f1.docx]

Supporting Information

**Single molecular semi-sliding ferroelectricity/multiferroicity**

Tingting Zhong^a^,* Hong Zhang^a^, Menghao Wu^b^ *

^a^ Department of Physics, Zhejiang Sci-Tech University, Hangzhou, Zhejiang 310018, China

^b^ School of Physics, Huazhong University of Science and Technology, Wuhan, Hubei 430074, China


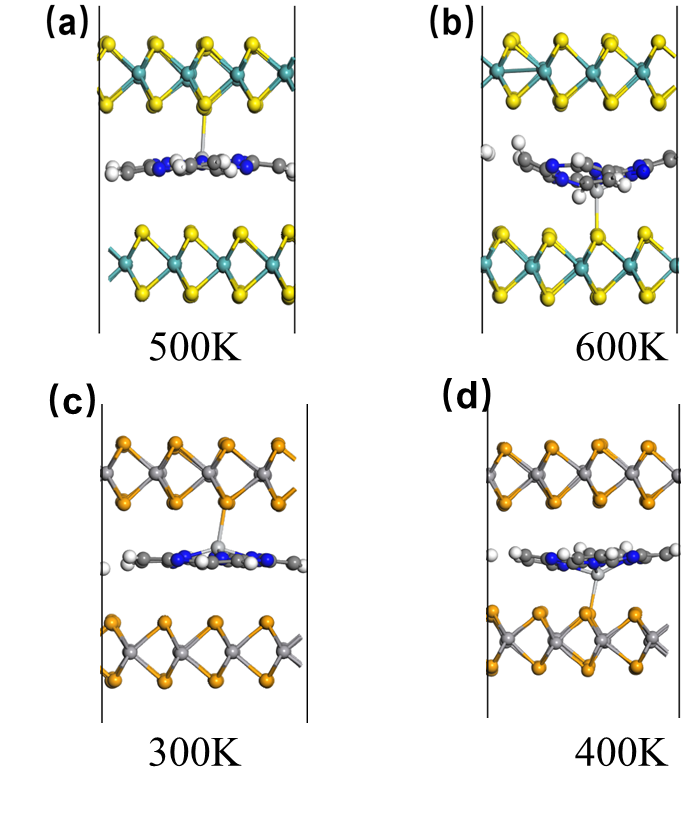


Figure S1. Snapshots of the equilibrium structures at (a) 500K and (b) 600K for bilayer MoS_2_ with intercalation of TiP molecules, (c)300K and (d)400K bilayer VSe_2_ with intercalation of TiP molecules, at the end of 6 ps of AIMD simulations with a canonical ensemble (time step is set as 1fs). Initially the Ti ions are bonded with the upper layer, and the flipping of anchor sites during the MD simulations in (b) and (d) may indicate the upper limit of Curie temperature.


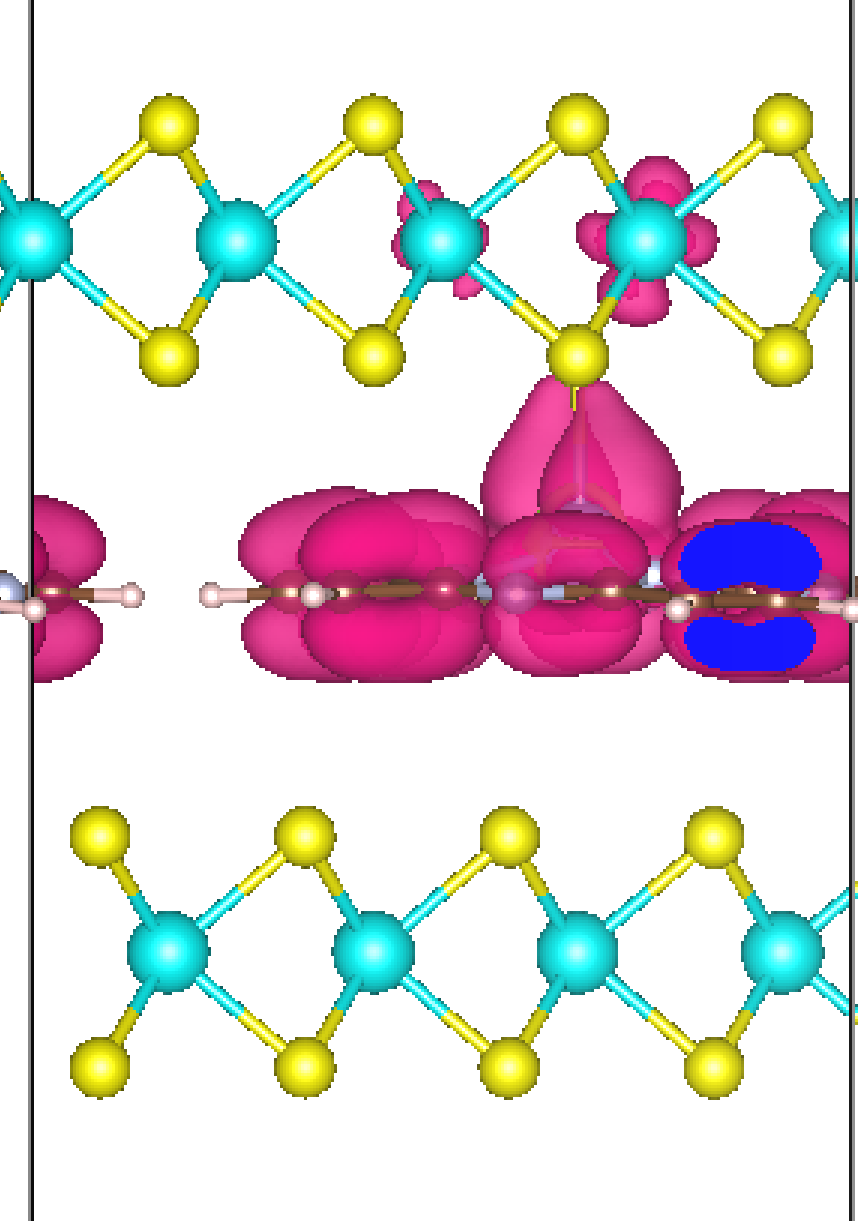

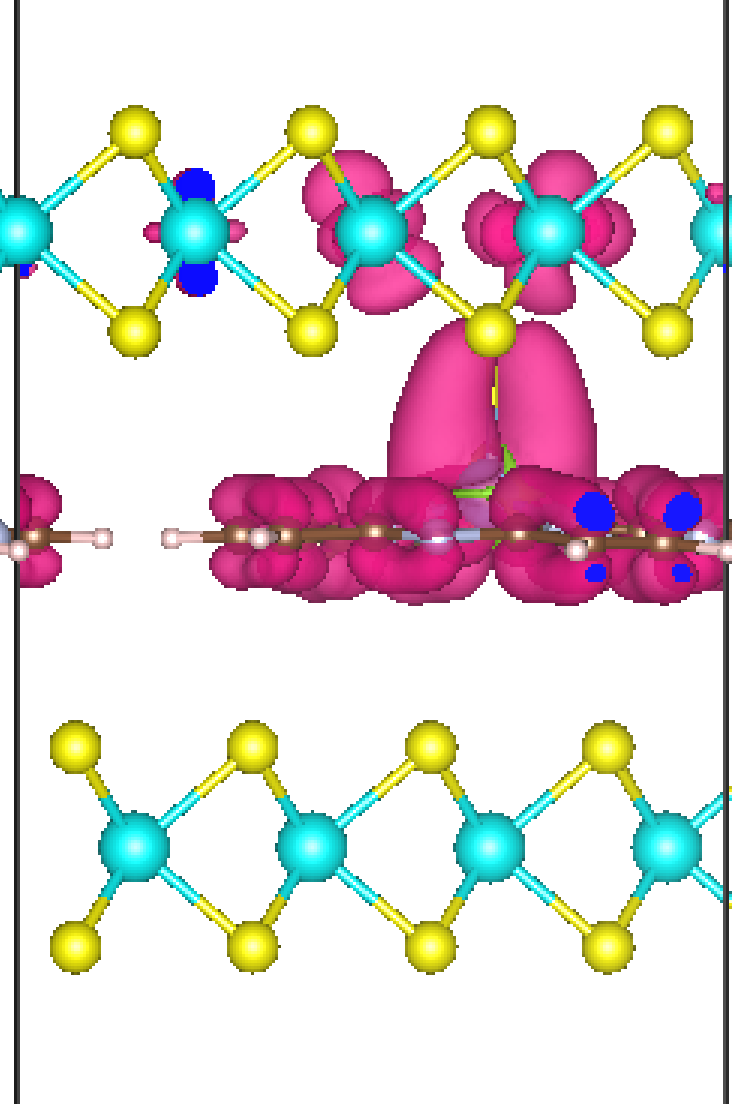


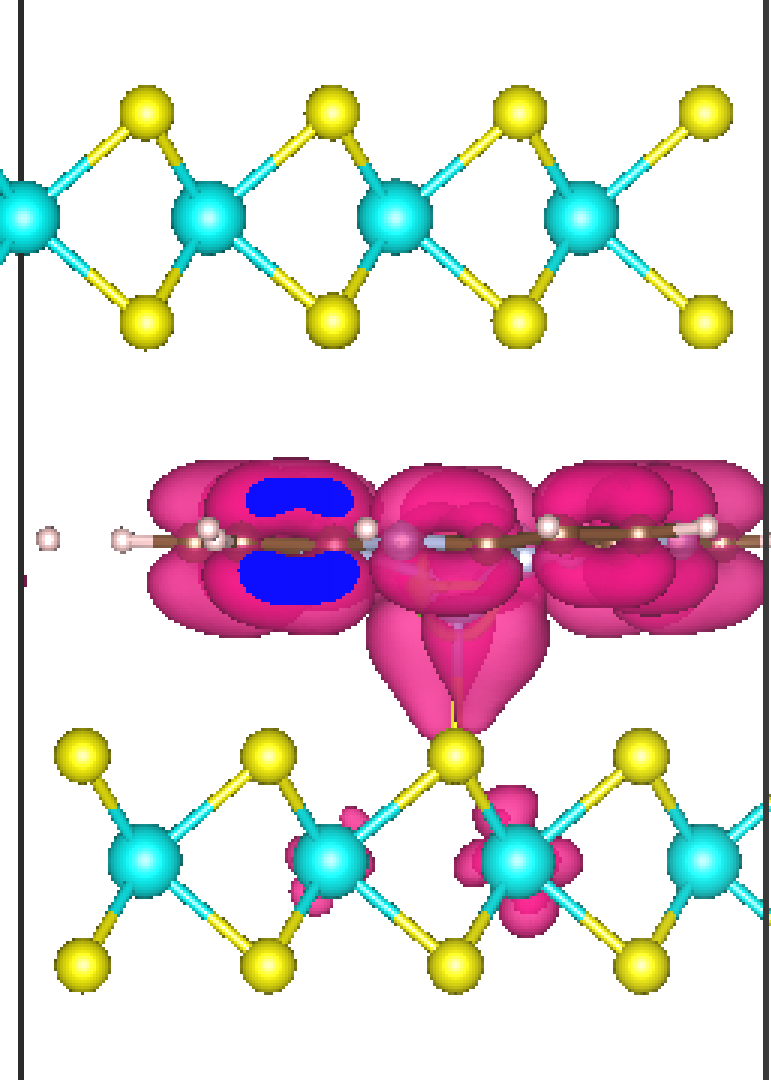

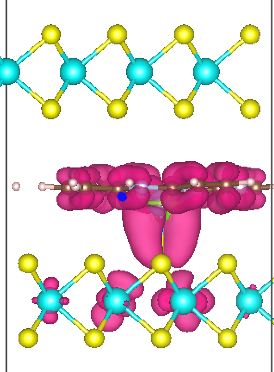


VBM CBM

Figure S2. The evolution of VBM and CBM distribution upon ferroelectric switching calculated by using HSE functional.
